# A Retrospective Cohort Study of Early Prosthetic Joint Infections at a US Academic Medical Center (2014-2023)

**DOI:** 10.7759/cureus.87744

**Published:** 2025-07-11

**Authors:** Grace D Cullen, Jorge L Salinas, Daisuke Furukawa

**Affiliations:** 1 Division of Infectious Diseases and Geographic Medicine, Stanford University School of Medicine, Palo Alto, USA

**Keywords:** bone and joint infections, early prosthetic joint infections, prosthetic hip infections, prosthetic joint infection (pji), prosthetic knee infections

## Abstract

Background

Prosthetic joint infections (PJIs) are rare but serious complications of hip and knee arthroplasties, associated with high morbidity and substantial healthcare costs. PJIs that occur within the first three months of surgery are considered “early” PJIs. We investigated the incidence, risk factors, and microbiology associated with early PJIs among patients undergoing hip or knee arthroplasties at Stanford Hospital.

Methods

A retrospective cohort study was conducted using data from the Centers for Disease Control’s (CDC) National Healthcare Safety Network (NHSN) for Stanford Hospital from 2014 to 2023. The study included adults who underwent hip or knee arthroplasties and developed early PJI. Potential PJI risk factors, including surgery type, age, sex, American Society of Anesthesiologists (ASA) score, diabetes status, body mass index (BMI), and the duration of surgery, were selected for analysis. Generalized estimating equations (GEE) were used to calculate adjusted odds ratios (aORs) for the development of early PJIs.

Results

Out of 13,197 surgeries, 119 cases of early PJIs were identified, with a cumulative incidence of 0.90%. Significant risk factors for early PJI included revision surgery (aOR: 2.44), male sex (aOR: 2.18), obesity (BMI of ≥30 kg/m²) (aOR: 1.57), ASA score of >2 (aOR: 1.66), and prolonged duration of surgery (aOR: 1.05 per 10-minute increment). Notably, hip arthroplasties were associated with a higher risk of early PJI compared to knee arthroplasties (aOR: 1.55). The most common causative pathogens were Gram-positive bacteria, making up 74.8% of cases. PJIs caused by Gram-negative organisms were associated with significantly longer index surgery durations compared to those caused by Gram-positive organisms (201 versus 153 minutes, p = 0.03).

Discussion

The findings align with existing literature that identifies revision surgery, male sex, obesity, ASA score, and surgical duration as risk factors for early PJIs. The higher incidence of PJIs in hip arthroplasties compared to knee arthroplasties may be influenced by underlying patient characteristics or surgical complexities. The microbiological profile revealed a predominance of Gram-positive organisms, and prolonged surgical time was associated with Gram-negative infections.

Conclusion

In this study, we report a relatively low incidence rate of early PJIs following hip and knee arthroplasties at our institution and identify key risk factors of PJIs, including revision surgery, male sex, obesity, ASA score, and surgical duration. Understanding these factors can aid in risk stratification and inform preoperative optimization strategies to reduce the incidence of PJIs in this patient population.

## Introduction

Prosthetic joint infection (PJI) is a rare but devastating complication following hip and knee arthroplasties, leading to increased morbidity, prolonged hospital stays, and substantial healthcare costs [1]. The incidence of PJIs varies between around 0.5% and 2% [2-8]. However, with the aging population in the United States, we can expect to see an increasing number of PJIs associated with significant healthcare costs [9]. PJIs are categorized as early, occurring within three months of surgery; delayed onset, occurring within 3-12 months of surgery; and late onset, occurring >12 months from surgery [1,10,11]. The risk of PJI is highest within the first two years of surgery [1,11]. Understanding the risk factors associated with early PJIs is essential for developing effective prevention strategies and improving patient outcomes.

Several demographic and clinical factors have been identified as potential risk factors for early PJIs, including younger patient age, male sex, obesity, American Society of Anesthesiologists (ASA) score of >2, immunosuppression, diabetes, the duration of surgery, and post-surgical complications such as hematoma or poor wound healing [1-4,6-8,11-18]. The most common microbiological cause of early PJIs is *Staphylococcus aureus* [1,16,17]. While less common, Gram-negative infections make up to 25% of all PJIs and are associated with worse outcomes compared to Gram-positive infections, with high rates of treatment failure [19]. With the most critical of the World Health Organization’s “Priority Pathogen” list consisting of Gram-negatives due to their ability to develop extensive drug resistance, these organisms pose a rising healthcare threat [20,21].

The primary objective of this study was to investigate the incidence, risk factors, and microbiology of early PJIs among patients undergoing hip and knee arthroplasties at Stanford Hospital from 2014 to 2023. As a secondary objective, we were particularly interested in rates of Gram-negative organisms and differences in patient and surgical factors between cases with infections due to Gram-negative compared to Gram-positive organisms. Utilizing data from the Centers for Disease Control’s (CDC) National Healthcare Safety Network (NHSN), we sought to identify risk factors associated with early PJIs of hip and knee arthroplasties. By elucidating these relationships, we hope to contribute to the existing body of knowledge and inform clinical practices aimed at reducing the incidence of PJIs in this patient population.

## Materials and methods

The study design was a retrospective cohort study. The Stanford University Human Subjects Research Institutional Review Board issued approval IRB-67967. The patient population included adults undergoing prosthetic knee or hip surgery for any indication at Stanford Hospital between January 1, 2014, and June 21, 2023, with the outcome of developing an early PJI. The dataset used was from the Centers for Disease Control’s National Healthcare Safety Network (NHSN). NHSN is the most widely used healthcare-associated infection tracking system in the United States and collects data from healthcare facilities nationwide [22]. Surgeries with the procedure codes “HPRO,” indicating arthroplasty of the hip, and “KPRO,” indicating arthroplasty of the knee, were identified from a dataset containing all surgical procedures (Dataset 1). HPRO and KPRO were further classified by subtype, including total primary (TOTPRIM), partial primary (PARTPRIM), total revision (TOTREV), or partial revision (PARTREV) surgery. From a second dataset containing all cases of surgical site infections (SSI) (Dataset 2), patients were identified as having a PJI by the specific event type descriptor “PJI.” These datasets were merged, joined by patient medical record number (MRN), procedure type, and the date of the index procedure for further analysis. Since the dataset included all infections arising within 90 days of index surgery, we assumed that all early PJIs during this time period were captured in this dataset.

Patients with the specific event type for “SIP” (superficial incision primary) were excluded from the analysis. A chart review was performed for patients with the specific event descriptors “BONE” (osteomyelitis) and “DIP” (deep incisional primary) to look for evidence of a diagnosis of early PJI. Surgical notes, infectious diseases consult notes, and microbiology corresponding to the event date were reviewed to identify if these patients met the criteria per the Infectious Diseases Society of America guidelines for PJI to be included in the final analysis [10]. The data were queried for missing or not available (NA) values, and when possible, chart review was used to identify the correct value. Mean imputation was used when the true value could not be identified. Mean imputation was chosen over more complex methods, such as multiple imputation, because only seven (0.05%) body mass index (BMI) values were missing, all from non-cases. Given this negligible proportion and the assumption of missingness being completely at random, mean imputation was considered an appropriate and efficient method, unlikely to meaningfully affect the results. The following variables were chosen for analysis as potential risk factors for PJI based on the literature and completeness of the dataset: procedure, procedure subtype, patient age at the time of procedure, sex, ASA score, diabetes, BMI, and the duration of surgery.

All statistics were performed using the statistical software RStudio version 2024.12.1+563 (Posit PBC, Boston, MA). Cumulative and annual incidences of early PJI were calculated. Median values with interquartile ranges (IQRs) were calculated for non-normally distributed continuous and ordinal variables, while proportions were calculated for categorical variables. Pearson’s chi-square test was used to compare proportions of categorical values. The nonparametric Wilcoxon rank-sum test was used to compare the median of the ordinal variable ASA and non-normally distributed continuous variables. Fisher’s exact test was used when categorial data sample size was five or less. Unadjusted odds ratios (ORs) and 95% confidence intervals were calculated for the following predictor variables with PJI as the outcome variable: procedure type (binary: KPRO versus HPRO), procedure subtype (binary: primary versus revision), age at the time of procedure (continuous), sex (binary: male versus female), ASA score category (binary: ≤2 versus >2), diabetes (binary: yes versus no), obesity status (binary: BMI of <30 versus ≥30), and the duration of surgery in 10-minute increments (continuous). Adjusted odds ratios (aORs) were calculated using generalized estimating equations (GEE) to analyze the impact of various predictors on the occurrence of early PJI. A sensitivity analysis (not shown) using different correlation structures (e.g., independent, exchangeable, autoregressive, and unstructured) was performed, and quasi-likelihoods under the independence model criterion (QICs) were compared to assess GEE model fit. An independent correlation structure was selected as the best-fitting model.

## Results

Subjects

**Figure 1:**
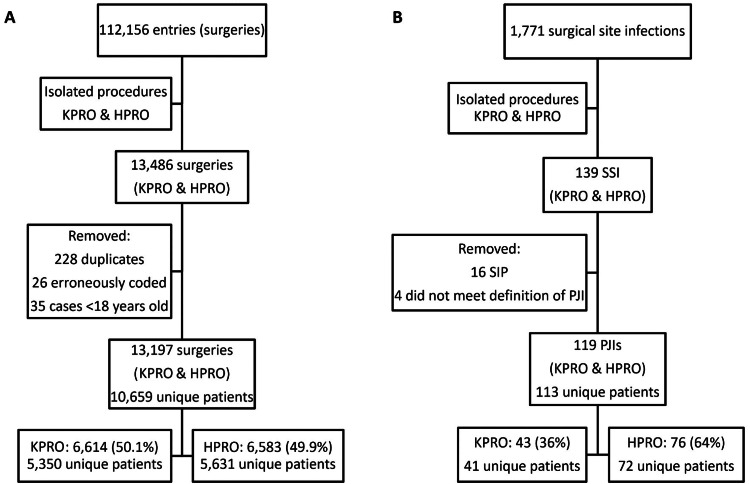
Identification of cases and non-cases using two NHSN datasets. Dataset 1 (A) includes all surgeries, and Dataset 2 (B) includes only surgical site infections (SSI). KPRO, prosthetic knee surgery; HPRO, prosthetic hip surgery; SIP, superficial incision primary; NHSN, National Healthcare Safety Network; PJI, prosthetic joint infection

Between January 1, 2014, and June 21, 2023, there were a total of 112,156 surgeries. Isolating for procedures KPRO and HPRO, there were a total of 13,197 surgeries, with 6,614 (50.1%) knee prosthetic surgeries and 6,583 (49.9%) hip prosthetic surgeries (Figure 1). Of these, 139 developed SSI with 119 early PJIs (43, knee; 76, hip), with an incidence of 0.90% or 9.0 PJIs per 1,000 surgeries (0.65%, knee; 1.15%, hip). Annual incidence ranged from 0.47% to 1.48%. There was no significant difference in cumulative incidence between the first half of the dataset (January 1, 2014, to December 31, 2018) and the latter (January 1, 2019, to June 21, 2023) (0.81% versus 1.01%, p = 0.3). A total of 2,199 (20.6%) patients had more than one surgery during this time period, with a median of one surgery (range: 1-10). Among patients with more than one surgery, the median was 2 (range: 2-10). Six (5.3%) patients with PJI had more than one occurrence of early PJI.

Non-cases had a greater proportion of total primary arthroplasties compared to cases of early PJI (70.4% versus 44.5%, p < 0.001), with early PJIs having a greater proportion of total revision surgeries (35.3% versus 14.1%, p < 0.001) (Table 1). The median age of patients with PJI was 65 (IQR: 60-73) compared to 67 (IQR: 59-75) in non-PJI patients, although this was not statistically significant. The majority of patients with PJI were men, while fewer than half of the non-PJI patients were men. At the time of index surgery, patients who developed PJI had an overall higher ASA score with a median of 3 (IQR: 2-3), compared to 2 (IQR: 2-3) among non-PJI patients. Patients in both groups had similar rates of diabetes, but the median BMI was higher in the PJI group. Patients who developed PJI had a longer duration of index surgeries with a median of 158 minutes (IQR: 116-232) compared to a median of 107 minutes (IQR: 86-137) in the non-PJI group.

**Table 1:** Patient and surgical characteristics of cases of early PJIs compared to non-cases. *P < 0.05 is considered significant. ^1^Test statistic: χ² = chi-square test; W = Wilcoxon rank-sum test. ^2^Pearson’s chi-square test. ^3^Wilcoxon rank-sum test. IQR, interquartile range; ASA, American Society of Anesthesiologists; BMI, body mass index; HPRO, prosthetic hip surgery; KPRO, prosthetic knee surgery; PJI, prosthetic joint infection

	Cases of Early PJI	Non-cases	P Value*	Test Statistic^1^
	N = 119	N = 13,078		
Sex (male), n (%)	77 (64.7)	5,591 (42.8)	<0.001^2^	22.31
Age, median (IQR)	65 (60-73)	67 (59-75)	0.12^3^	842,475
Procedure, n (%)	HPRO: 76 (64)	HPRO: 6,507 (50)	0.003^2^	8.84
	KPRO: 43 (36)	KPRO: 6,571 (50)		
Procedure subtype, n (%)			<0.001^2^	53.08
Total primary	53 (44.5)	9,210 (70.4)		
Total revision	42 (35.3)	1,846 (14.1)		
Partial primary	7 (14.3)	912 (7.0)		
Partial revision	17 (14.3)	1,110 (8.5)		
Revision (any)	59 (49.6)	2,956 (22.6)		
ASA, median (IQR)	3 (2-3)	2 (2-3)	<0.001^3^	652,653
Duration in minutes, median (IQR)	158 (116-232)	107 (86-137)	<0.001^3^	415,767
BMI in kg/m^2^, median (IQR)	30.2 (25.7-34.9)	28.4 (24.8-33.0)	0.01^3^	670,070
Diabetes, n (%)	23 (19.3)	2,098 (16)	0.40^2^	0.72

GEE model

Unadjusted odds ratios were calculated for each variable as a predictor of early PJI (Table 2). Patients with early PJIs had a 78% increase in odds of having had a prosthetic hip surgery compared to patients who had a prosthetic knee surgery. A revision surgery resulted in over three times an increase in odds of developing early PJI. Men had a nearly 2.5 times increase in odds of early PJI compared to women. Patients with an ASA score of greater than 2 had twice the odds of developing a PJI compared to patients with a score of 2 or less. Longer duration and obesity also had statistically significant higher odds ratios among patients with early PJI. The unadjusted OR for age and diabetes was not statistically significant. After adjusting for other variables, the analysis revealed that procedure type (prosthetic hip surgery), revision surgery, male sex, ASA score of greater than 2, the increased duration of the index surgery, and obesity all contributed to increased odds of early PJI, although odds ratios were attenuated after adjustment.

**Table 2:** Unadjusted and adjusted odds ratios (ORs) of risk factors for the development of PJI. *GEE used to calculate adjusted odds ratios. GEE, generalized estimating equations; HPRO, prosthetic hip surgery; ASA, American Society of Anesthesiologists; BMI, body mass index; CI, confidence interval; PJI, prosthetic joint infection

	Unadjusted OR (95% CI)	Adjusted OR (95% CI)*
Procedure (HPRO)	1.78 (1.23-2.62)	1.55 (1.02-2.35)
Revision procedure	3.37 (2.34-4.84)	2.44 (1.67-3.58)
Age	0.99 (0.98-1.00)	1.00 (0.98-1.01)
Sex (male)	2.46 (1.69-3.61)	2.18 (1.47-3.24)
ASA > 2	2.01 (1.39-2.95)	1.66 (1.08-2.56)
Duration (10-minute increments)	1.07 (1.05-1.08)	1.05 (1.04-1.07)
Obesity (BMI ≥ 30 kg/m^2^)	1.59 (1.11-2.28)	1.57 (1.04-2.39)
Diabetes	1.25 (0.78-1.94)	1.07 (0.67-1.72)

Knee subset

Among patients with early PJIs of prosthetic knees, patients were more likely to be younger and male than those without infection (Table 3). Similar to the results of the combined PJI dataset, patients with knee PJIs had higher proportions of revision surgeries, higher median ASA scores, and longer median durations of index surgery. In this subset, however, patients did not have significant differences in BMI or increased odds of obesity or diabetes. A GEE model was not performed due to the small sample size and concerns for overfitting the model.

**Table 3:** Patient and surgical characteristics of cases of early PJIs of knee arthroplasties compared to non-cases. *P < 0.05 is considered significant. ^1^Test statistic: χ² = chi-square test; W = Wilcoxon rank-sum test. ^2^Logistic regression used to estimate unadjusted odds ratios (ORs). ^3^Fisher’s exact test. ^4^Pearson’s chi-square test. ^5^Wilcoxon rank-sum test. PJI, prosthetic joint infection; CI, confidence interval; NA, not applicable; IQR, interquartile range; ASA, American Society of Anesthesiologists; BMI, body mass index

	Cases of Early PJI	Non-cases	P Value*	Test Statistic^1^	Unadjusted OR (95% CI)^2^
	N = 43	N = 6,571			
Procedure subtype, n (%)					
Total primary	22 (51.2)	5,146 (78.3)	<0.001^3^	NA	Reference
Total revision	13 (30.2)	575 (8.8)			5.29 (2.58-10.41)
Partial primary	3 (7.0)	310 (4.7)			2.26 (0.53-6.58)
Partial revision	5 (11.6)	540 (8.2)			2.17 (0.72-5.31)
Revision (any)	18 (41.9)	1,115 (17.0)			3.52 (1.89-6.45)
Sex (male), n (%)	29 (67.4)	2,610 (39.7)	<0.001^4^	12.56	3.14 (1.69-6.14)
Age, median (IQR)	63 (58.5-69)	68 (61-74)	0.006^5^	175,491	0.96 (0.94-0.98)
ASA, median (IQR)	3 (2-3)	2 (2-3)	0.05^5^	119,500	ASA > 2
					2.05 (1.11-3.94)
Duration in minutes, median (IQR)	138 (106-196)	106 (85-132)	<0.001^5^	89,360	Duration (10-minute increments)
					1.06 (1.03-1.08)
BMI in kg/m^2^, median (IQR)	29.4 (27.0-35.1)	29.8 (26.2-34.3)	0.73^5^	136,991	Obesity (BMI ≥ 30 kg/m^2^)
					0.84 (0.45-1.52)
Diabetes, n (%)	9 (20.9)	1,262 (19.2)	0.93^4^	0.01	1.11 (0.50-2.23)

Hip subset

Among patients with early PJIs of prosthetic hips, patients were more likely to be male than those without infection but were similar in age (Table 4). Similar to the results of the combined PJI dataset, patients with hip PJIs had a greater proportion of revision surgeries, higher median ASA scores, higher median BMI, and longer median durations of index surgery. In this subset, patients did not have a significant difference in rate or increased odds of diabetes.

**Table 4:** Patient and surgical characteristics of cases of early PJIs of hip arthroplasties compared to non-cases. *P < 0.05 is considered significant. ^1^Test statistic: χ² = chi-square test; W = Wilcoxon rank-sum test. ^2^Logistic regression used to estimate unadjusted odds ratios (ORs). ^3^Pearson’s chi-square test. ^4^Wilcoxon rank-sum test. PJI, prosthetic joint infection; CI, confidence interval; IQR, interquartile range; ASA, American Society of Anesthesiologists; BMI, body mass index

	Cases of Early PJI	Non-cases	P Value*	Test Statistic^1^	Unadjusted OR (95% CI)^2^
	N = 76	N = 6,507			
Procedure subtype, n (%)					
Total primary	31 (40.8)	4,064 (62.5)	<0.001^3^	24.36	Reference
Total revision	29 (38.2)	1,271 (19.5)			2.99 (1.79-4.99)
Partial primary	4 (5.3)	602 (9.3)			0.87 (0.26-2.21)
Partial revision	12 (15.8)	570 (8.8)			2.76 (1.36-5.30)
Revision (any)	41 (53.9)	1,841 (28.3)			2.97 (1.89-4.70)
Sex (male), n (%)	48 (63.2)	2,981 (45.8)	0.004^3^	8.41	2.03 (1.28-3.28)
Age, median (IQR)	66.5 (60-75)	66 (57-75)	0.82^4^	243,581	1.00 (0.99-1.02)
ASA, median (IQR)	3 (2-3)	2 (2-3)	0.006^4^	206,370	ASA > 2
					1.98 (1.25-3.22)
Duration in minutes, median (IQR)	180 (129-254)	109 (87-144)	<0.001^4^	121,893	Duration (10-minute increments)
					1.07 (1.05-1.08)
BMI in kg/m^2^, median (IQR)	30.7 (25.5-34.8)	27.0 (23.7-31.2)	<0.001^4^	186,515	Obesity (BMI ≥ 30 kg/m^2^)
					2.75 (1.75-4.37)
Diabetes, n (%)	14 (18.4)	836 (12.8)	0.20^3^	1.61	1.53 (0.82-2.67)

Microbiology

**Figure 2:**
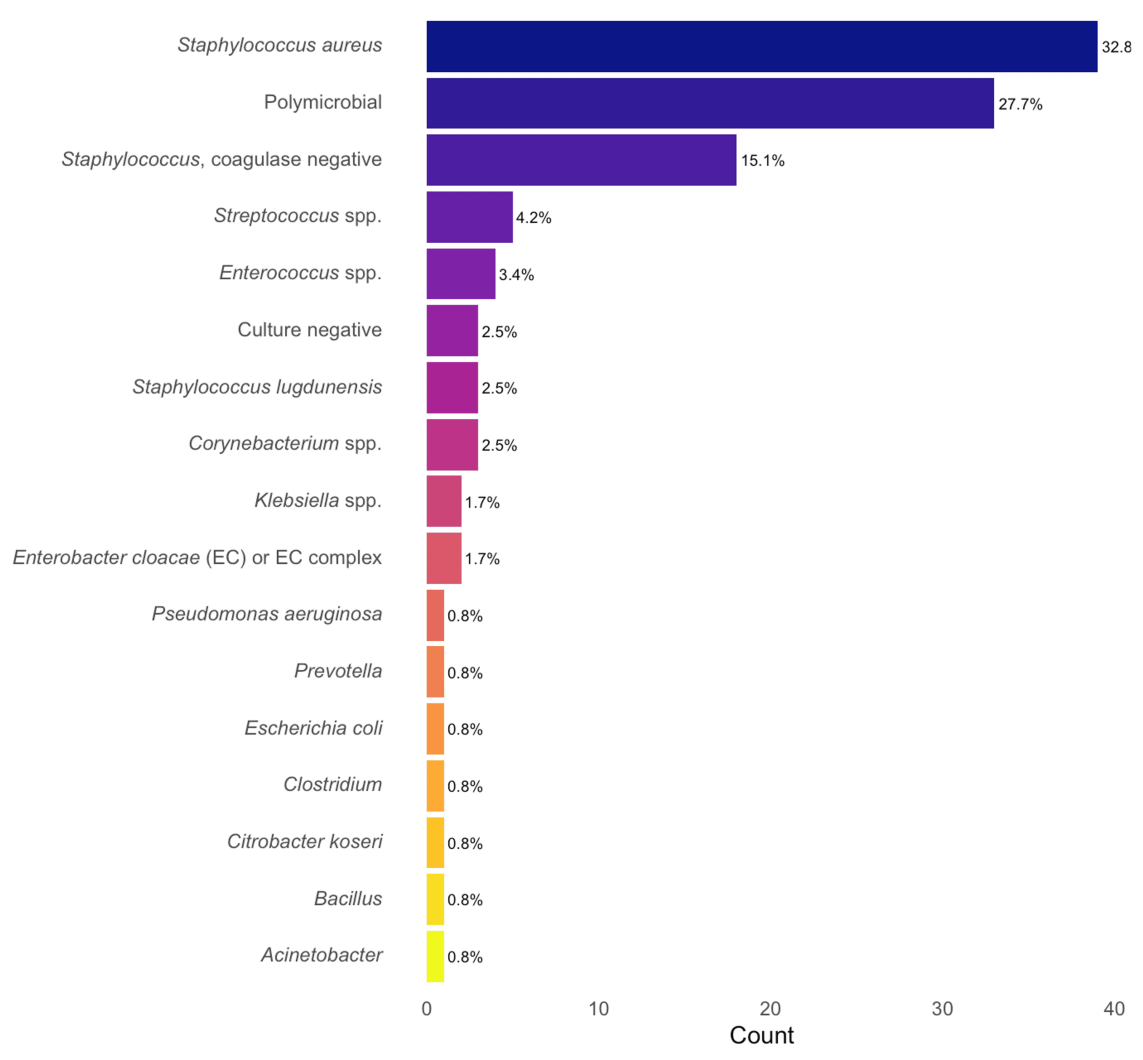
Infectious causes of PJIs by patient case. PJIs: prosthetic joint infections

The majority (74.8%, 89/119) of infections were due to Gram-positive organisms, compared to 22.7% (27/119) of Gram-negative and 2.5% (3/119) of culture-negative (Table 5). About a third of patients had infections due to *Staphylococcus aureus* alone, with nearly 30% of patients having polymicrobial infections (two or more pathogens) (Figure 2). Coagulase-negative staphylococci (CoNS) were the second most frequently isolated bacteria overall, followed by *Enterococcus *and *Streptococcus *species. However, in monomicrobial infections, *Streptococcus *species were more frequently isolated than *Enterococcus *(Figure 2). Gram-negative pathogens were overall uncommon. *Escherichia coli* was the most frequently isolated Gram-negative organism when including polymicrobial infections, whereas *Klebsiella* species and *Enterobacter cloacae *complex were the most common in monomicrobial infections. Yeast (*Candida *species) was also rare and only isolated with other bacteria in polymicrobial infections. Gram-negative infections were more frequently part of polymicrobial infections with 66.7% (18/27), compared to 9.8% (9/92) of Gram-positive infections (Table 5). Patients with infections due to Gram-negative organisms were more likely to have longer surgeries, but there were no significant differences between groups for other factors.

**Table 5:** Patient and surgical characteristic comparison between Gram-negative and Gram-positive/other PJIs. *P < 0.05 is considered significant. ^1^Test statistic: χ² = chi-square test; W = Wilcoxon rank-sum test. ^2^Unadjusted OR estimated with logistic regression. ^3^Pearson’s chi-squared test. ^4^Wilcoxon rank-sum test. PJIs, prosthetic joint infections; CI, confidence interval; IQR, interquartile range; ASA, American Society of Anesthesiologists; BMI, body mass index; OR, odds ratio

	Gram-Positive and Culture-Negative	Gram-Negative	P Value*	Test Statistic^1^	Unadjusted OR (95% CI)^2^
	N = 92 (77.3)	N = 27 (22.7)			
Polymicrobial infection, n (%)	9 (9.8)	18 (66.7)	<0.001^3^	23.97	10.3 (4.00-28.34)
Sex (male), n (%)	61 (66.3)	16 (59.3)	0.66^3^	0.2	0.74 (0.31-1.82)
						Age, median (IQR)	65 (59.6-70.4)	69 (60.5-77.5)	0.57^4^	1,331	1.01 (0.98-1.05)
Procedure, n (%)	HPRO: 55 (59.8)	HPRO: 21 (77.8)	0.14^3^	2.2	0.43 (0.14-1.10)
	KPRO: 37 (40.2)	KPRO: 6 (22.2)			
Procedure subtype, n (%)	Primary: 48 (52.2)	Primary: 12 (44.4)	0.63^3^	0.24	1.36 (0.58-3.28)
	Revision: 44 (47.8)	Revision: 15 (55.6)			
ASA, median (IQR)	3 (2-3)	3 (2-3)	0.36^4^	1,367	1.53 (0.75-3.39)
Duration in minutes, median (IQR)	153 (98-207)	201 (111-291)	0.03^4^	1,595	1.01 (1.00-1.01)
BMI in kg/m^2^, median (IQR)	29.7 (25.3-34.1)	33.0 (27.5-38.5)	0.80^4^	1,283	1.00 (0.93-1.06)
Diabetes, n (%)	19 (20.7)	4 (14.8)	0.69^3^	0.16	0.67 (0.18-2.00)

## Discussion

This study investigated the risk of early PJI among patients who underwent hip or knee arthroplasty between 2014 and 2023 at our institution using an NHSN dataset. The incidence of early PJI was low and similar to other studies [5,15,16]. We found that revision surgery, male sex, obesity (BMI ≥ 30 kg/m^2^), ASA score of >2, and the prolonged duration of surgery were risk factors of early PJI, which several other studies have concluded [1-3,7,13,15,23-27]. The mechanism for why male sex is a risk factor for PJI is not well-understood, but it has been suggested that anatomical differences or differences in activity level between sexes could explain this risk [26,27]. Obesity is a recognized modifiable risk factor for infection and other postoperative complications, for reasons previously described [28]. ASA score of >2, which can be taken as a surrogate for overall patient health and fitness, is also an expected risk factor [3,13,15,25-27]. While we were unable to characterize patients’ individual comorbidities from this dataset, the higher median ASA score among the patients with early PJI can be taken to mean that this patient population was sicker at baseline overall. Longer duration of surgery is also a previously identified risk factor for PJI [3,11,13,26,27]. The complexity of the surgery or intraoperative complications likely increases surgical time. While we were not able to know the underlying indication for each surgery from the dataset, from the chart review of a few of the longer surgeries, it was found that some of these patients had tumors requiring multidisciplinary surgical management, extending the operating time. Our study did not find age or diabetes to be risk factors. However, similar to other studies, our patients with PJIs tended to be younger, although this difference did not reach statistical significance [7,13]. While diabetes has been identified as a risk factor for PJI, it is possible that in our cohort, where most arthroplasties were likely to be elective, patients selected for surgery had well-controlled diabetes [6,13,18,25]. We did not have fasting glucose or hemoglobin A1c (HA1c) values at the time of surgery to know the severity of the patients’ diabetes, although one study suggests HA1c may not be a useful predictive biomarker of infection risk [29].

In our study, we found a greater risk of PJI among patients with hip arthroplasties compared to knee. Prior studies have shown a higher incidence of PJI among knee arthroplasties compared to hip [1,2,6,9]. It has been suggested that the greater mobility of the knee joint and less soft tissue coverage may explain why knee arthroplasties have greater rates of infection than hip arthroplasties [1,12]. However, a large Dutch study that included 171,512 arthroplasties from a national database found a PJI incidence of 1.2% among total hip arthroplasties (THAs) and 0.7% among total knee arthroplasties (TKAs), similar to incidences found in our study [16,30]. One noticeable difference between the hip and knee arthroplasty subsets is that in our cohort, patients who developed hip PJIs tended to have higher BMIs compared to those who did not develop infection. In the knee subset, we did not see a significant difference in BMI between those with PJI and those without infection, which may reflect limited power to detect an association within this subgroup due to small sample size. Unfortunately, we do not have further patient information from our dataset to understand why more patients with hip arthroplasties compared to knee had greater rates of early PJIs. Although this is speculation, it is possible that this patient population, from a large academic tertiary referral center, had an overrepresentation of uncommon indications for arthroplasty such as bone tumor, resulting in selection bias or higher rates of post-surgical complications such as hematomas, a known infection risk [2,11].

The microbiology epidemiology at our institution is similar to other reports, with *Staphylococcus aureus* the leading cause of early PJI in our cohort, followed by CoNS [1,16]. We were interested in the patient and surgery characteristics among patients with infections caused by less common, Gram-negative pathogens. In our cohort, we had a similar rate of Gram-negative infections to other studies [4,17]. Many Gram-negative infections were part of polymicrobial infections, which have implications for antimicrobial therapy. Patients with polymicrobial infections may require more than one antibiotic, especially in the setting of increasing antimicrobial resistance, which not only increases cost but also increases a patient’s risk for medication side effects. There were no significant patient differences between those with infections due to Gram-positive versus Gram-negative organisms. However, surgical duration was significantly longer in the Gram-negative group. One likely explanation is that longer surgical duration increases the risk of intraoperative contamination, as the wound remains open to the environment for a longer period, allowing more opportunity for environmental or Gram-negative bacteria to enter the surgical field. Alternatively, the longer length of surgery could be explained by the complexity of the case, which could in part be due to underlying patient comorbid factors that may place them at higher risk for infection. The total number of PJIs due to Gram-negative pathogens was too small to perform further analysis using multivariable regression modeling to estimate adjusted odds ratios.

The strengths of this study include the design using a retrospective cohort study as opposed to a case-control study, making use of the entire surgical population at our institution during the study period. We expect that this NHSN dataset was comprehensive in capturing all early PJIs, and the data for the selected variables were complete with very little missingness. Since PJI is a very rare event, we can approximate relative risk to odds ratios.

There are several important limitations to this study. First, it was an observational study, so conclusions of causation cannot be made. These data were subject to reporting bias and coding errors, which may have led to the misclassification of both the exposure and the outcome. The dataset was limited with regard to patient details and did not include demographic information, such as race or socioeconomic status, or specific comorbidities beyond diabetes and obesity. Indication, an important potential risk factor, was not captured in the dataset. Therefore, for example, a patient who underwent a revision surgery for late PJI could not be distinguished from a patient who had revision surgery for a noninfectious indication. Previous PJI is a known risk factor for recurrent PJI, but we were unable to evaluate this in our study [18]. Additionally, we did not have information on patient outcome data with regard to treatment success or need for revision surgery due to recurrent PJI. We were unable to perform pathogen-specific subgroup analyses due to small sample size or assess antimicrobial resistance patterns since these were not captured in our dataset. These are important factors that can influence treatment outcomes in PJIs and warrant further investigation in future studies.

## Conclusions

Our retrospective cohort study identified incidence, risk factors, and microbiology associated with early PJIs among patients undergoing hip and knee arthroplasties at a large academic referral center over nearly a 10-year period. These findings align with existing literature, reinforcing the importance of these variables in predicting infection risk. Potentially addressable risk factors (e.g., weight management and the optimization of comorbidities) reflected in higher ASA scores present perioperative opportunities to reduce infection risk. The microbiological profile of our cohort demonstrated a predominance of Gram-positive organisms, particularly *Staphylococcus aureus*, which is consistent with previous reports. Future prospective studies including outcome data would help clarify differences in patient characteristics and the clinical severity of patients with Gram-positive versus Gram-negative PJIs. Overall, this study contributes valuable insights into the epidemiology of early PJIs following hip and knee arthroplasties, emphasizing the need for targeted preoperative assessments and interventions.
